# The impact of daily activities on the mental health of older adults in Mexico

**DOI:** 10.3389/fpsyg.2026.1951484

**Published:** 2026-09-28

**Authors:** Diana Betancourt Ocampo, Patricia Andrade Palos, Alejandro González-González

**Affiliations:** 1 Faculty of Psychology, Universidad Anáhuac México, México City, Mexico; 2 Faculty of Psychology, Universidad Nacional Autónoma de México, México City, Mexico

**Keywords:** aging, depression, mental health, older adults, subjective well-being

## Abstract

**Objective:**

This study analyzed the role that daily activities (goals and plans, healthy routines, social connections, healthy thinking, and meaningful activities) play in relation to two indicators of mental health (depressive symptoms and subjective well-being) among older adults.

**Methods:**

A non-probability sample of 1,018 residents of Mexico City (23.3% men and 76.7% women) aged 60 to 97 years (*M* = 74.97, SD = 7.60) was selected. The Things You Do Questionnaire (TYDQ-21) was used to assess daily activities, along with the adapted version of the Yesavage Geriatric Depression Scale (GDS) and the Subjective Well-Being Scale (EBS-8).

**Results:**

The results showed that 19% of the participants exhibited symptoms of depression. Significant correlations were found among all variables, and multiple regression analyses indicated that most dimensions of daily activities were predictors of depressive symptoms (explaining 13% of the variance) and subjective well-being (explaining 20% of the variance).

**Conclusion:**

The findings of this study suggest that the daily activities assessed in this research could be taken into account when developing interventions that promote mental health in older adults. The results of this study provide empirical evidence that may be useful for developing mental health promotion programs for older adults based on everyday activities that do not necessarily require the involvement of specialists.

## 1 Introduction

The World Health Organization (WHO) reports that in 2019, it was estimated that one in eight people worldwide lived with a mental disorder, meaning that 970 million people worldwide lived with such conditions, which are more common in women (52.4%) than in men (47.6%). In addition, the WHO reports that anxiety and depression are the most common conditions, accounting for 31 and 28.9%, respectively. Although the presence of certain mental disorders may vary throughout the life cycle, anxiety and depression are conditions that remain relatively constant from adulthood onward (age 20 and older), and they are more common in women than in men (World Health Organization, 2022).

Despite mental health being a global issue, in Mexico there are additional factors that complicate the timely provision of care for these types of problems; for example, wait times are longer compared to those for physical health conditions (Berenzon et al., 2013), and at the government level, there is insufficient investment for its care (Barrón-Velázquez et al., 2024; Berenzon et al., 2013; Díaz-Castro et al., 2020), which may be contributing to a shortage of mental health specialists in the public sector and an uneven distribution of those who are available across the country’s different states (Barrón-Velázquez et al., 2024). Furthermore, there are population groups that require increasing levels of care due to their steady growth, such as older adults; according to the Consejo Nacional de Población (National Population Council) the aging index has increased over the past 30 years (Secretaría General del Consejo Nacional de Población (CONAPO), 2025), which implies that a greater number of mental health professionals will be needed to provide care to this population group.

Regarding data on this population, the WHO reports that 14% of adults aged 70 and older worldwide live with a mental disorder, with depression and anxiety being the most common conditions (World Health Organization, 2026). According to data from the Encuesta Nacional sobre Salud y Envejecimiento en Mexico (National Survey on Health and Aging in Mexico, ENASEM, by its Spanish acronym), a higher percentage of women report five or more symptoms of depression (31.3% for the 60–69 age group, 34.9% for women aged 70–79, and 42.3% for those aged 80 and older) in contrast to men (18.1% for the 60–69 age group, 20.8% for the 70–79 age group, and 29.8% for those aged 80 and older); furthermore, the results indicate that these rates increase with age (Instituto Nacional de Estadística y Geografía, 2026).

Depression is defined as a negative emotional state that can range from unhappiness and discontent to an extreme sense of sadness, pessimism, and discouragement, and that often interferes with daily life. It is often associated with changes in eating habits and sleep patterns, a lack of energy or motivation, difficulty concentrating or making decisions, and withdrawal from social activities (American Psychological Association, 2026). As people age, the various changes that older adults face, such as chronic health conditions, the loss of loved ones, and the inability to perform activities they were once able to do, may give rise to negative emotions linked to symptoms of depression. According to the literature, this could increase the mortality rate; therefore, it is important to address this issue in this population group (American Psychological Association, 2022).

Although symptoms of depression are clearly a recurring indicator of mental health, some authors suggest that the absence of these symptoms does not necessarily imply optimal mental health; therefore, concepts such as subjective well-being have been incorporated to provide a broader understanding of psychological distress. Diener et al. (1999) define subjective well-being as people’s evaluation of their own lives, which includes three main aspects: (1) life satisfaction, (2) positive affect, and (3) negative affect. The ENASEM (Instituto Nacional de Estadística y Geografía, 2026) found high levels of life satisfaction among Mexicans aged 50 and older, with slightly higher prevalence rates among men compared to women; for example, when asked about their life satisfaction, 89.1% of men reported being satisfied, while in the case of women, it was 84.3%. These findings are interesting, as one might expect that, given that the proportion of older adults in our country exhibiting symptoms of depression ranges from 20 to 40%, the number of older adults reporting satisfaction with their lives would be much lower. In this regard, some studies indicate that both can coexist; that is, there may be people with mild or moderate depressive symptoms who simultaneously report a high level of subjective well-being. For this reason, these studies suggest that both indicators should be included when assessing well-being. In this regard, some studies indicate that the two can coexist; that is, there may be people with mild or moderate depressive symptoms who simultaneously report a high level of subjective well-being. For this reason, these studies suggest that both indicators should be included when assessing mental health (Keyes, 2005, 2007).

Some authors (Bisby et al., 2023; Bisby et al., 2022; Titov et al., 2022) indicate that conditions such as depression and anxiety are characterized by certain behavioral and cognitive patterns that contribute to their onset and maintenance, and that these patterns can be modified in the pursuit to reduce symptoms. For this reason, certain studies have set out to identify actions or activities in daily life that can contribute to the treatment of certain conditions and the promotion of health. For example, Santini et al. (2022) identified beliefs and actions associated with good mental health in the adult population (aged 18 and older) in Denmark; the authors found a total of 92 categories, which they grouped into five broad themes: social relationships (e.g., social life, talking with others), healthy behavior (e.g., exercising, eating healthily), relaxation (e.g., stress reduction), positive mindset (e.g., optimism), and brain engagement (e.g., playing games such as crossword puzzles/sudoku).

Terides et al. (2016) conducted a study in which they designed an instrument to assess the frequency of both adaptive behaviors and cognitions associated with therapeutic change from the perspective of cognitive-behavioral theory; this tool consisted of four dimensions: cognitive restructuring, social interactions, rewarding behaviors, and activity planning. The researchers also assessed symptoms of depression, anxiety, and life satisfaction and found that participants who reported more adaptive behaviors and cognitions were those who exhibited fewer depressive and anxiety symptoms, as well as higher scores on life satisfaction.

On the other hand, there is the work of Titov et al. (2022) who conducted a literature review to identify those everyday activities that could promote mental health, as the authors note that, although there is information about these actions beneficial for mental health, it was still unknown which ones were the most important, what impact they might have on mental health indicators, and how frequently they are performed. With the information obtained, the research team compiled a bank of items comprising more than 500 activities related to psychological well-being, derived from psychological therapies and healthy habits; they then filtered these items and developed an assessment tool (Things You Do Questionnaire, TYDQ) that initially consisted of 96 items. The instrument was applied to an adult population (including older adults), and the results of the factor analyses (exploratory and confirmatory) yielded a shortened version with 21 items (TYDQ-21), which were grouped into five categories: meaningful activities (activities that provide a sense of accomplishment, satisfaction, and joy), goals and plans (aspects that contribute to motivation), healthy thinking (realistic thoughts about oneself, the world, and the future), healthy routines (daily activities such as sleeping, eating properly, and staying active), and social connections (regular contact with close others to foster a sense of belonging). In addition, the authors report negative associations between nearly all dimensions of the instrument (except for social connections) with depressive and anxiety symptoms; they also found positive correlations with life satisfaction in four of the five dimensions, except for the goals and plans dimension.

Bisby et al. (2022) conducted a study examining the relationship between daily activities and psychological distress in Canadian adults and older adults who received online therapy. The authors confirmed the five-factor structure of the TYDQ-21 in a Canadian population; furthermore, they found that daily activities mediated treatment-related change in depressive and anxiety symptoms. They also found that participants who reported engaging in daily activities with high frequency were those who reported fewer depressive and anxiety symptoms. In another study (Bisby et al., 2023), treatment-related change in the frequency of daily activities assessed using the TYDQ-21 was analyzed. The authors provided a group of adults (including older adults) with access to an Internet-based cognitive-behavioral therapy program; these participants also completed assessments of depression and/or anxiety symptoms. The results showed that the structure of the TYDQ-21 was confirmed in this sample, and significant correlations were found in both pre- and post-treatment assessments between the total score for daily activities (obtained using the TYDQ-21) and symptoms of depression, anxiety, and life satisfaction, where participants with higher scores on daily activities exhibited fewer symptoms of both anxiety and depression and achieved higher scores on life satisfaction.

Other studies have set out to analyze the effect of daily activities based on the framework proposed by Titov et al. regarding clinical trials (Bisby M. A. et al., 2024;Bisby M. et al., 2024; Hadjistavropoulos et al., 2025; Titov et al., 2024), and although they have used different types of study designs, in general terms the results have shown that daily activities may be a mechanism associated with the onset, maintenance, and reduction of symptoms of both depression and anxiety.

As can be seen, daily activities as proposed by Titov and colleagues have provided empirical evidence on how goals and plans, healthy routines, social connections, healthy thoughts, and meaningful activities are related to better mental health in the adult population, including older adults; however, it is important to note that, while some of these dimensions may seem similar to those assessed in other instruments for older adults, such as the WHO-QOL-OLD (World Health Organization, 2006), the overarching concepts they measure are of a different nature. The WHO-QOL-OLD (World Health Organization, 2006) was designed to assess quality of life in older adults, whereas the approach proposed by Titov et al. (2022) with the TYDQ-21 assesses the frequency with which everyday behaviors and actions are related to mental health.

Although several studies that have examined daily activities have included older adults in their samples, it would be important to examine whether this framework could be applied in the same way to a sample of older adults in Mexico. For this reason, the present study examined whether the dimensions of daily activities explain certain aspects of mental health assessed using two indicators: depressive symptoms and subjective well-being, in a sample of older adults in Mexico.

## 2 Materials and methods

### 2.1 Participants

A non-probabilistic sample of 1,018 older adults residing in the 16 boroughs of Mexico City was selected; 23.3% were men and 76.7% were women, ranging in age from 60 to 97 years (*M* = 74.97, SD = 7.60). As shown in Table 1, the largest percentage of participants reported having a primary school education, being married, living with other family members, being a homemaker, and having a monthly income of $3,000 to $4,999.

**Table 1:** Distribution of participants by sociodemographic variables.

Characteristics	*n*	%
Education level		
No education	119	11.7
Primary school	383	37.6
Secondary school	214	21.0
High school	90	8.8
Technical degree	120	11.8
Bachelor’s degree	87	8.5
Postgraduate	5	0.5
Marital status		
Married	376	36.9
Widowed	351	34.5
Single	169	16.6
Divorced	96	9.4
Cohabiting	26	2.6
Living arrangements		
With other relatives	479	47.1
With spouse/partner	326	32.0
Alone	173	17.0
With other people	40	3.9
Occupation		
Household	577	56.7
Retired	287	28.2
Self-employed	60	5.9
Unemployed	51	5.0
Employee	43	4.2
Income		
Do not know	132	13.0
Up to $1,499 mexican pesos	130	12.8
$1,500 to $2,999 mexican pesos	213	20.9
$3,000 to $4,999 mexican pesos	289	28.4
$5,000 to $7,999 mexican pesos	157	15.4
$8,000 or more mexican pesos	97	9.5

### 2.2 Measures

To assess daily activities that promote mental health, the “Things You Do Questionnaire” (TYDQ-21) by Titov et al. (2022) was used. This instrument assesses five dimensions: Goals and Plans (5 items), Healthy Routines (3 items), Social Connections (3 items), Healthy Thinking (6 items), and Meaningful Activities (4 items); it uses a Likert-type scale with five response options (ranging from “Never” to “Always”). This instrument has been shown to have adequate psychometric properties (Bisby et al., 2022; Titov et al., 2022); the authors report a total reliability (Cronbach’s alpha) of 0.900 for the instrument. For the purposes of this research, four researchers participating in the study adapted the instrument into Spanish, who independently translated and adapted the items and then reviewed each item to finalize the version that was applied.

On the other hand, to measure depression, the adapted version for the Mexican population (Salgado et al., 2024) of the Yesavage Geriatric Depression Scale (GDS; Yesavage et al., 1983) was applied. This is a unidimensional instrument consisting of 14 dichotomous (yes/no) items, and in the Mexican population, it yielded a reliability coefficient of 0.94. The Subjective Well-Being Scale (EBS-8) by Calleja and Mason (2020) was also used; it measures life satisfaction and positive affect and consists of eight Likert-type items with six response options (from “strongly disagree” to “strongly agree”), This instrument was developed for the Mexican population (including older adults) and has demonstrated empirical evidence of validity and reliability (α = 0.958).

To identify certain sociodemographic characteristics of the participants, a questionnaire with eight closed-ended questions was used (e.g., sex, age, education level, occupation). In addition, the Mini-Cog (Borson et al., 2003) was used to assess whether older adults had the cognitive capacity to complete the instruments; the version administered included a set of three words to recall and a task to draw a clock set to 11:10.

### 2.3 Procedure

This project received support from the Instituto para el Envejecimiento Digno (Institute for Dignified Aging, [INED]) in Mexico City, whose staff, trained to provide care for older adults, participated in the data collection process. To this end, they were instructed on how to administer the survey. INED staff administered the paper-and-pencil instruments to older adults who visit some of its centers (spaces where older adults can share experiences and engage in physical and mental activities), where they were informed of the study’s objective and asked for their voluntary participation. They were also told that the information collected was confidential and intended for research purposes. For those older adults who agreed to participate, the questionnaires were administered in the form of individual interviews, to have fewer missing or invalid responses (de Waal et al., 2012; Smeeth et al., 2001). It should be noted that, prior to administering the instruments, participants were given the Mini-Cog to assess whether they possessed the basic cognitive ability required to understand the test items; only those participants who recalled two or three words were given the remaining instruments (Borson et al., 2003).

### 2.4 Data analysis

Descriptive analyses (e.g., frequencies, percentages, mean, standard deviation) were conducted to identify the sociodemographic characteristics of the participants. To assess the validity of the instruments used, confirmatory factor analyses (CFA) were conducted, using the maximum likelihood method, in which the following criteria were considered to determine whether the model had a good fit (Hu and Bentler, 1999): (1) the *χ*^2^/d*f* ratio (CMIN/DF), which should have a value <3; (2) the CFI (Comparative Fit Index) and TLI (Tucker-Lewis Index), with values >0.90; (3) the SRMR (Root Mean Square Residual) with a value <0.05; (4) the RMSEA (Root Mean Square Error of Approximation) with a value <0.08. For the GDS and EBS-8, the models tested were one-dimensional; for the TYDQ-21, the goal was to validate a five-dimensional model. The reliability of the scales was calculated using Cronbach’s alpha and McDonald’s omega (*ω*) coefficient (Stefana et al., 2025).

To conduct comparative tests regarding symptoms of depression and subjective well-being by sex, Student’s *t*-tests for independent samples were performed; however, since the sample size for women (*n* = 729) was larger than that for men (*n* = 226), a random subsample of 30% was drawn from the women, resulting in groups of equivalent size in terms of the number of participants; it was with this subsample that the comparisons were made.

Furthermore, based on the depression scale score (Instituto Nacional de Geriatría, 2020), the total sample of older adults was categorized into two groups: those without symptoms (0 to 4 points) and those with depressive symptoms (5 or more points); since these groups differed significantly in the number of participants, a random subsample (23%) was selected from the group of older adults classified as having no symptoms of depression for the comparative analyses (Student’s *t*-tests for independent samples). This ensured two groups of similar size, and the comparative analyses were performed using this subsample, which sought to determine statistically significant differences in the dimensions of the TYDQ-21 and subjective well-being between older adults with and without symptoms of depression. Cohen’s d was calculated to assess the effect size of the differences (Cohen, 1988).

Subsequently, using the complete sample, Pearson correlations were calculated between the dimensions of daily activities (TYDQ-21) and depressive symptoms and subjective well-being. This was followed by multiple linear regression analyses in which the five dimensions of daily activities were included as independent variables and depressive symptoms and subjective well-being as dependent variables. Data processing and analysis were performed using Jamovi (version 2.3.15.0) and SPSS (version 24). For all analyses, a level of statistical significance of *α* = 0.05 was considered.

### 2.5 Ethical considerations

All principles of the Declaration of Helsinki were followed. Participants were informed about the study’s objectives, the purpose of the questions, the voluntary nature of participation, and the institutions involved in conducting the study. Informed consent was also obtained from each participant. The study was approved by the Ethics Committee of Anahuac University in Mexico.

## 3 Results

Table 2 shows the AFC results for the scales used. As can be seen, both the depression scale (GDS) and the subjective well-being scale (EBS-8) obtained adequate fit indices for all their items, as well as satisfactory reliability values. In the case of the TYDQ-21, adequate fit indices were obtained after removing one item from the healthy thinking dimension (“You treated yourself with respect”); the reliability value for the entire instrument was satisfactory (Table 2). The reliability for each of the dimensions was as follows: goals and plans, *α* = 0.854, *ω* = 0.854; healthy routines, *ω* = 0.621, *ω* = 0.622; social connections, *α* = 0.710, *ω* = 0.717; healthy thinking, *α* = 0.738, *ω* = 0.746; meaningful activities, *α* = 0.802, *ω* = 0.803.

**Table 2:** Instrument fit and reliability indices.

Measure	*χ*^2^ (gl)	*p*	CMIN	CFI	TLI	RMSEA (CI)	*α*	*ω*
GDS	3548.39 (91)	0.001	38.99	0.905	0.887	0.059 (0.054–0.065)	0.765	0.784
EBS-8	134.86 (19)	0.001	7.09	1.00	1.00	0.067 (0.056–0.977)	0.971	0.972
TYDQ-21	924 (160)	0.001	5.77	0.921	0.906	0.067 (0.063–0.071)	0.929	0.931

When comparing the subsample of women with the group of men in terms of symptoms of depression (*t* = 0.229, *p* > 0.050) and subjective well-being (*t* = 0.805, *p* > 0.050), the results showed that there were no statistically significant differences, that is, both men and women had similar mean scores for both depressive symptoms and subjective well-being. Based on the GDS cutoff score (five or more points), 19% of older adults exhibited depressive symptoms. When comparing older adults with and without symptoms of depression across the five dimensions of daily activities (TYDQ-21) and subjective well-being, the results indicated statistically significant differences (Table 3). Older adults with symptoms of depression had lower mean scores on daily activities as well as on subjective well-being compared to those without symptoms. Overall, the effect size was moderate, except in the case of subjective well-being, where a large effect was found (Cohen, 1988).

**Table 3:** Comparison of mean values for dimensions of daily activities and subjective well-being among older adults with and without symptoms of depression.

Variable	No symptoms of depression (*n =* 191)		With symptoms of depression (*n =* 181)		*t*	*p*	*d*
	*M*	DE	*M*	DE			
Goals and plans	2.65	1.05	1.98	1.27	5.52	<0.001	0.57
Healthy routines	3.19	0.73	2.56	1.07	6.61	<0.001	0.68
Social connections	2.81	0.90	2.21	1.19	5.49	<0.001	0.56
Healthy thinking	2.54	0.92	2.03	1.20	4.60	<0.001	0.47
Meaningful activities	2.85	0.91	2.13	1.19	6.52	<0.001	0.67
Subjective well-being	40.30	7.59	30.02	10.27	10.83	<0.001	1.13

The score range for the TYDQ-21 dimensions is 0 to 4 points; for subjective well-being, it is 8 to 48.

Table 4 presents the results regarding the correlations between scores on daily activities, symptoms of depression, and subjective well-being; as can be seen, significant correlations were found among all the variables, with older adults who engage in daily activities more frequently exhibiting fewer symptoms of depression and reporting greater subjective well-being. In addition, it was found that symptoms of depression were negatively associated with subjective well-being.

**Table 4:** Correlation coefficients between daily activities, depressive symptoms, and subjective well-being.

Variable	1	2	3	4	5	6	7
1. Goals and plans	—	0.567**	0.631**	0.748**	0.821**	−0.326**	0.410**
2. Healthy routines		—	0.495**	0.510**	0.579**	−0.288**	0.276**
3. Social connections			—	0.588**	0.715**	−0.270**	0.342**
4. Healthy thinking				—	0.699**	−0.236**	0.376**
5. Meaningful activities					—	−0.341**	0.433**
6. Depressive symptoms						—	−0.465**
7. Subjective well-being							—

**p < 0.001.

Regarding the effect of the dimensions of the Daily Activities Instrument (TYDQ-21) on depressive symptoms, the findings indicated that the “Meaningful Activities” dimension was included in the first step of the equation [*F*(1, 953) = 125.73, *p <* 0.001]; in the second step, the “Healthy Routines” dimension was added [*F*(1, 952) = 71.04, *p <* 0.001], and in a third step, the “Goals and Plans” dimension was added [*F*(1, 951) = 48.81, *p <* 0.05]; together, these dimensions explained 13% of the variance in depressive symptoms (Table 5).

**Table 5:** Effect of the TYDQ-21 dimensions on depressive symptoms.

Predictor	*B*	SE *B*	*β*	*R* ^2^	Δ*R*^2^
Step 1. Meaningful activities	−0.472**	0.138	−0.184	0.116	
Step 2. Healthy routines	−0.365**	0.109	−0.125	0.128	0.013
Step 3. Goals and plans	−0.251*	0.127	−0.106	0.131	0.004

**p < 0.001; *p < 0.05.

Table 6 shows the results regarding the effect of the dimensions of the daily activities instrument (TYDQ-21) on subjective well-being. The results showed that the “Meaningful Activities” dimension was included in the first step of the equation [*F* (1, 998) = 229.75, *p <* 0.001]; in the second step, the “Healthy Thinking” dimension was added [*F* (1, 997) = 122.58, *p* < 0.001], and in the third step, the “Goals and Plans” dimension was added [*F* (1, 996) = 83.44, *p <* 0.05], which together explained 20% of the variance in subjective well-being among older adults.

**Table 6:** Effect of the TYDQ-21 dimensions on subjective well-being.

Predictor	*B*	SE *B*	*β*	*R* ^2^	Δ*R*^2^
Step 1. Meaningful activities	2.49**	0.478	0.266	0.186	
Step 2. Healthy thinking	0.977*	0.418	0.103	0.196	0.010
Step 3. Goals and plans	0.992*	0.476	0.114	0.198	0.003

**p < 0.001; *p < 0.05.

## 4 Discussion

Nowadays, it is undeniable that the older adult population is constantly growing and that, in a few years, healthcare services will be insufficient, especially when it comes to mental health. For this reason, the objective of this study was to identify the role played by five types of daily activities on two indicators of mental health: symptoms of depression and subjective well-being, in a sample of older adults. In general terms, the results showed that older adults who engage in meaningful activities, have healthy thinking and routines, as well as goals and plans were those who exhibited fewer symptoms of depression and greater subjective well-being.

The results of this research showed that no significant differences were found between men and women in either depression symptom scores or subjective well-being. These findings are interesting because the latest national data (National Institute of Statistics and Geography, 2026) suggest that women experience more symptoms of depression than men, a trend that also increases with age (Instituto Nacional de Estadística y Geografía, 2026). This discrepancy in the results may be due to the fact that the sample of older adults in this study generally showed low scores on depression symptoms; even when classified as having or not having symptoms (which is the same criterion used by ENASEM), only one-fifth of the sample studied was found to have depression symptoms.

In addition to this, the data from this study showed that participants scored high on subjective well-being and that these scores were similar among men and women, information consistent with that reported in the 2024 ENASEM, which indicates that the population over 50 years of age exhibited high levels of life satisfaction (one of the dimensions of subjective well-being) and that, although the results of this national survey suggest higher scores among men than among women, there is no mention of statistically significant differences. Furthermore, this study found a significant negative relationship between subjective well-being and symptoms of depression, a finding that was somewhat expected and confirms that including these two indicators to assess mental health could be valuable; however, it would be interesting for future studies to carefully analyze the coexistence of these indicators among older adults in our country to confirm Keyes’s (2005, 2007) proposal.

This study found that older adults with symptoms of depression engage less frequently in the assessed daily activities and reported lower levels of subjective well-being. These findings may confirm the role of daily activities (goals and plans, healthy routines, social connections, healthy thinking, and meaningful activities) in the presence of symptoms of depression, which, to some extent, supports the findings of Titov et al. (2022) that such activities can promote mental health. This is consistent with the findings of various authors who report on the role that various daily activities play in promoting, maintaining, and increasing levels of life satisfaction, quality of life, and well-being, as well as a negative association with depressive symptoms and/or emotional distress (Adams et al., 2011; Jarosz, 2022; Kavčič and Avsec, 2018; Menec, 2003).

Similarly, the results of the regression analyses showed that most dimensions of daily activities (except for the dimension of social connections) were predictors of both depressive symptoms and subjective well-being. The “meaningful activities” dimension refers to actions that provide a sense of accomplishment, satisfaction, or joy, which may be simple yet rewarding activities, and was the most significant predictor in both regression analyses. However, while previous authors have reported significant associations between this dimension and similar indicators of mental health (Adams et al., 2011; Bisby et al., 2022; Bisby et al., 2023; Titov et al., 2022), some of whom have included this dimension as part of the total score for the “daily activities” variable. It is important to keep in mind that the older adults from whom data were collected attend community centers where they engage in activities that may be meaningful to them, which could partially explain the results, since these centers promote interaction, socialization, and recreational and occupational activities, excluding therapeutic or health-care interventions (Talmage et al., 2020).

According to Titov et al. (2022), having goals and plans could help people stay motivated, as they provide a sense of hope and prevent people from dwelling solely on the past. In this study, the “goals and plans” dimension was also a predictor of both depressive symptoms and subjective well-being, which does not entirely align with the findings of Titov et al. (2022), where this dimension did not show a significant relationship with life satisfaction but did show a relationship with depression and anxiety in the adult population; however, the findings of that study involved older adults. While having goals and plans may be important at any stage of life, older adults may experience a reorganization of their life plans as a result of changes in health, occupation, and social roles; thus, goals can provide direction, purpose, and meaning in life, which becomes particularly important when the external demands that previously structured older adults’ lives diminish or change (Barlow et al., 2020).

In this study, the only dimension of daily activities that was not included as a predictor variable was that of social connections. This could be because most of the older adults assessed do not live alone, which would imply that they have frequent contact with people who are important to them; therefore, there may be other factors that are more significant in explaining both subjective well-being and symptoms of depression, though this does not mean that the role of social ties, interaction, and socialization, which are fundamental for older adults, is not recognized, as there is evidence that limited or absent social contact impacts the physical and mental health of older adults (Hajek et al., 2025; Nakou et al., 2025).

While this study provides interesting findings on certain daily activities that promote mental health in older adults, it is important to note that the results cannot be generalized given the nature of the participant selection. On the one hand, the sample was not randomly selected; and although data were collected from older adults in different regions of Mexico City, these participants generally took part in social, cultural, and physical activities organized by a government agency, a factor that may characterize this sample. Therefore, it is recommended that future studies be conducted to corroborate these findings among older adults who are not part of these programs. It would also be important to conduct research with a similar number of male and female participants, which could help determine whether the role of daily activities varies by gender.

Finally, it is important to highlight the need to generate evidence regarding the role that daily activities play in the mental health of older adults, and to recognize that, far from being structured interventions, promoting these activities is much more viable and feasible, given that many systematic interventions pose a challenge in terms of adherence and maintenance, as people often find them too complicated and inaccessible. Therefore, having these “everyday” alternatives represents a significant advantage for the well-being of this population.

## Data Availability

The raw data supporting the conclusions of this article will be made available by the authors, without undue reservation.
